# Association of Military Aircraft Noise Exposure with Mental Well-Being and Sleep Disturbance near a Military Air Base in Okinawa, Japan: An Ecological Study

**DOI:** 10.3390/ijerph23010054

**Published:** 2025-12-31

**Authors:** Yuka Maekawa, Daisuke Nonaka, Sae Kawamoto, Yukako Maeda, Yuko Toyama

**Affiliations:** Graduate School of Health Sciences, University of the Ryukyus, Ginowana 901-2720, Japan

**Keywords:** aircraft noise, well-being, sleep disturbance, military base, Japan

## Abstract

A considerable number of people are exposed to noise from military aircraft daily, but its health effects have not been sufficiently examined. This study assessed the association of exposure to such noise with mental well-being and sleep disturbance among people living in Okinawa prefecture, where there are two U.S. military air bases. In 2024, data were collected from 394 residents in high-, low-, and no-exposure communities using the WHO-5 Well-being Index and the Athens Insomnia Scale. Among respondents, 55.8% were female; the largest age groups were 70’s (25.4%) and 60’s (23.6%). Poor mental well-being and sleep disturbance were most prevalent in the high-exposure community (poor mental well-being: 38.2%, sleep disturbance: 46.6%), followed by low-exposure (36.1%, 46.3%) and no-exposure (21.9%, 29.0%) communities. Multivariate logistic regression analyses showed that compared to no-exposure community, the high-exposure and low-exposure communities were significantly more likely to have poor mental well-being (odds ratio (OR): 1.84, 95% confidence interval (CI): 1.05–3.23; OR: 1.94, 95% CI: 1.05–3.56), as well as sleep disturbance (OR: 1.98, 95% CI: 1.17–3.35; OR: 2.04; 95% CI: 1.16–3.59, respectively). The results suggest that there is a substantial need to address the noise from military aircraft in Okinawa.

## 1. Introduction

Globally, it is estimated that 36 million people were exposed to harmful levels of noise from civilian aircraft daily in 2018 [1]. However, this estimate likely understates the actual burdens of aircraft noise because it does not account for exposure to noise from military aircraft, which has not been studied sufficiently. Aircraft noise can cause a variety of problems related to physical health, mental health, and well-being, such as sleep disturbances, hypertension, cardiovascular diseases, cognitive impairment, annoyance, depression, and adverse birth outcomes [2,3,4,5,6,7]. The adverse health effects of noise from military aircraft is considered to be larger than those of civilian aircraft [8,9], but much fewer studies have assessed these effects [9].

There are 63 U.S. military facilities in Japan under the Treaty of Mutual Cooperation and Security (signed in 1960) between Japan and the U.S.A., and Japan’s southernmost prefecture, Okinawa, has 26 of these facilities [10], which is related to historical and geopolitical reasons. Two of the facilities in Okinawa are Futenma Air Station and Kadena Air Base. Kadena Air Base occupies the three municipalities of Kadena town, Yomitan village, and Okinawa city, including approximately 80% of Kadena’s land area [11]. The Okinawa prefectural government conducted a four-year research project in the late 1990s to assess the health effects of aircraft noise from Futenma Air Station and Kadena Air Base. The results showed that people living in the vicinity of these bases are significantly more likely to have related problems compared to those living far from the bases, which include depression, sleep disturbance, disturbance of daily activities, annoyance, poor life satisfaction, misbehavior of children, low birth weight, pre-term birth, and hearing impairment [12,13]. However, the findings of these studies are unlikely to be applied to the current situations because the current situations could be greatly different from the situations where these studies were conducted: Since the 1990s, the number of houses with soundproof measure has increased and the military aircrafts used in the base have changed.

The Kadena government conducted community-based surveys in 2006 and 2020 to assess residents’ perceptions of the adverse effects of Kadena Air Base using a self-developed questionnaire. Both surveys indicated that almost all respondents felt that the aircraft noise was loud or very loud and that they had been affected or badly affected as a result. The respondents complained about feelings of unwellness, annoyance, and irritation; sleep and hearing problems; and disturbances to their daily lives [14].

Well-being is highly valued by the Organization for Economic Co-operation and Development (OECD) as an indicator for evaluating social progress “beyond GDP”. In line with this, the Japanese government conducts a survey with municipalities to assess factors that potentially influence the well-being of community people. The environmental standard for noise is one of such factors. However, few studies have assessed the impact of noise from military aircraft on well-being and sleep disturbance using a validated, commonly used scale, which has compromised the validity and comparability of the findings globally.

Therefore, this study was conducted to answer the following question “is exposure to military aircraft noise associated with mental well-being and sleep disturbance among people living in the vicinity of Kadena Air Base?”.

## 2. Materials and Methods

### 2.1. Study Design, Site, and Sampling Procedures

**Figure 1:**
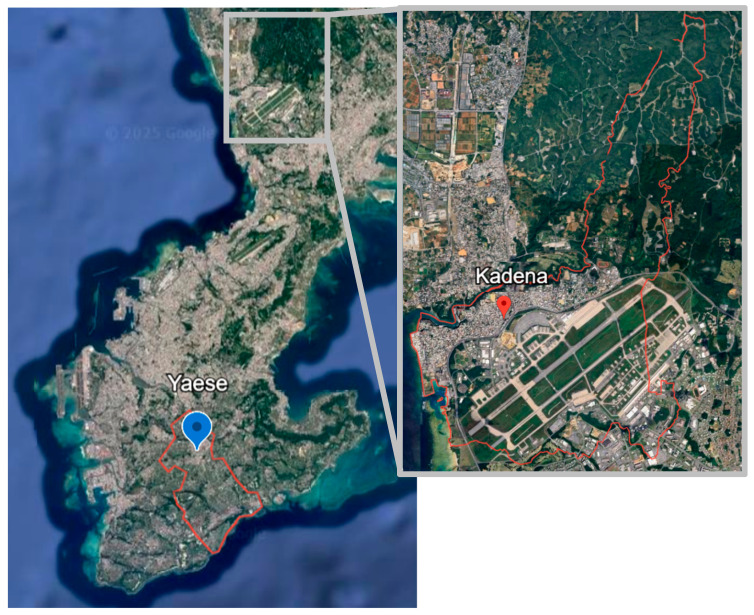
Town map of Kadena and Yaese. The area indicated by the red pin shows the town Kadena, which contained the high- and low-exposure communities. The area indicated by the blue pin shows the town Yaese, which contained the no-exposure community. The map was created by the authors using Google earth (URL https://earth.google.com/earth/d/1fGl6OoLincPxU15pQk6SYvKcZO7yh-Kp?usp=sharing) (accessed on 30 October 2025).

In this cross-sectional ecological study, the main explanatory variable was the exposure level of residential communities, which were assumed that people living in the same community were exposed to the same noise level. The outcome variables were mental well-being and sleep disturbance, which were measured at the individual level. This study was conducted in Kadena and Yaese in Okinawa prefecture (Figure 1). Kadena was selected as the exposure area, while Yaese was the no-exposure area.

Okinawa prefecture consists of 11 towns, 11 cities, and 19 villages. The town of Yaese was selected due to its equivalent municipal status to that of Kadena, and it is the least likely to be affected by noise from military bases due to the absence of aviation-related facilities in the surrounding area.

This study selected high-exposure and low-exposure communities from the six communities in Kadena according to data on outdoor environmental noise measured by the Okinawa prefectural government over the past five years (2020–2024) (See Supplementary File S1) [15,16,17], as well as the results of the 2020 community-based survey done by the Kadena town [14]. The high-exposure community had the highest annual average noise level, the most frequent annual average number of noise occurrences and the highest number of residents who reported the aircraft noise is very loud. For example, in 2024, it was 63 dBA (*L_den_*) and 70.6 noise occurrence per day. In contrast, the low-exposure community had the lowest annual average noise level, the lowest annual average number of noise occurrences and the lowest number of residents who reported the aircraft noise is very loud. For example, in 2024, it was 61 dBA (*L_den_*) and 27.5 noise occurrences per day.

The no-exposure community was selected from the 34 communities in Yaese, which was similar to the two selected communities in Kadena in terms of the residential environment characteristics. Household maps were obtained from the leaders of the communities and used to select several blocks to visit in each one. Around 220 households per community were visited (all households in the selected blocks).

### 2.2. Data Collection

Data were collected between March and December 2024 through a survey using self-administered questionnaires. Before the survey, residents were informed about this study by a leaflet inserted in the town letters. Survey teams visited all households in the selected blocks and tried to hand over the questionnaires in person. To enhance resident participation, the survey teams made two visits to households on a weekend or weekday. If it was unable to see any household members, the questionnaire in a return envelope was dropped in their mailbox.

The respondents were able to choose how they would submit the questionnaire from one of the following three methods: submission to the survey team, postal mail submission, and online submission. Overall, the survey teams visited 827 households and distributed a total of 1346 questionnaires. Ultimately, 394 responses were returned.

### 2.3. Variables and Measurements

Mental well-being as the primary outcome was measured using a validated Japanese version of the World Health Organization’s five-item Well-being Index (WHO-5-J) [18,19,20]. This brief mental health-assessment tool was developed by the WHO and has been translated into many languages [21,22]. It consists of five items that are each scored from 0 to 5, with higher total scores indicating better mental well-being. Total scores below 13 indicate poor mental well-being status and require further examination for the potential presence of a depressive disorder.

Sleep disturbance as the secondary outcome was assessed using a validated Japanese version of the Athens Insomnia Scale (AIS) [23]. The AIS is a self-administered instrument that assesses insomnia symptoms using the WHO’s International Classification of Diseases, 10th Revision (ICD-10) [24,25]. It consists of eight items that are rated on a four-point scale, and a total score ≥ 4 indicates suspected insomnia, while a score ≥ 6 indicates clinical insomnia. The exposure variable was the different noise levels of residential communities: high-exposure, low-exposure, and no-exposure. Covariates included socio-demographic and economic factors (sex, age, employment type, number of household members, and perceived economic situation), health-related factors (hearing ability, chronic illness, sleep medicine/supplements, and perceived level of noise), and lifestyle factors (daily hours at/near home, napping, coffee/tea consumption, alcohol consumption, smoking, physical activities per day, and housing type).

### 2.4. Statistical Analysis

Fisher’s exact test and logistic regression were used because the outcome variables were a binary variable (i.e., presence/absence of poor mental well-being and presence/absence of sleep disturbance). Due to the nature of the present study (i.e., ecological study design), no data was available on the individual-level noise exposure. Therefore, the analyses of the present study were performed on the assumption that people living in the same community were exposed to the same level of noise exposure. Fisher’s exact test was used in bivariate analyses to examine the association between the outcome variables and each of the exposure variables. The significance level was set at *p* < 0.05. Logistic regression was used to examine two models in multivariate analyses. Model 1 included the main exposure variable along with age and sex. In addition to these, model 2 also included the exposure variables that were found to be significantly associated with the outcome variable in the bivariate analyses. One exposure variable, perception of noise, was significantly associated with the outcome variables, but we did not include it in model 2 because of the strong association with the main exposure variable (i.e., collinearity). Analysis of non-respondents was not done as no information was available on the demographic characteristics of non-respondents. All analyses were performed using IBM SPSS Statistics version 24.

### 2.5. Ethical Considerations

Along with the self-administered questionnaires, explanation sheets about this study were distributed to all participants and they were required to read the explanation sheets. The responders then confirmed their consent to participate in this study by checking the consent box on the cover page of the questionnaire. Voluntary participation was ensured by enabling only individuals who opted to participate in this study to submit the questionnaire freely at a later time. This study was approved by the Ethics Committee for Medical and Health Research Involving Human Subjects of the University of the Ryukyus, Japan (Permit number: 23-2214-00-00-00, 8 December 2023).

## 3. Results

### 3.1. Participant Characteristics

There 394 participants in this study, of which 155 (39.3%) were from the no-exposure community, 108 (27.4%) were from the low-exposure community, and 131 (33.2%) were from the high-exposure community (Table 1). Approximately half of the participants (55.8%) were female, and the largest age group was 70–79 years (25.4%), followed by 60–69 years (23.6%). The most common employment type was regular employees (22.4%), followed by housewives/husbands (20.4%) and retirees (20.4%). The most common household size was 2 people (35.9%), followed by 4 or more (29.0%). The majority of participants (62.2%) perceived their economic situation as neither difficult nor comfortable, and less than one-third (27.2%) perceived their economic situation as difficult or very difficult.

Regarding the perceived level of noise, approximately one-fourth (26.9%) felt that it is very noisy around their homes. Most of the participants (68.5%) reported having no hearing difficulties. Among those who did report hearing difficulties, 11.3% used hearing aids. Less than one-fourth of the participants (20.4%) experienced insomnia or other sleep problems, and 36.5% of those participants were receiving treatment or under observation for the condition at the time of the survey. Some participants (13.6%) were using sleep medicines or supplements. Nearly two-thirds of the participants (62.6%) reported having at least one chronic illness, of which the most commonly reported was hypertension, followed by other cardiovascular diseases and chronic pain.

Most of the participants (74.4%) spent 12 h per day or more at or near their homes. The median bedtime was 22:30 (inter-quartile range: 21:30–23:30), and the median waking time was 6:00 (inter-quartile range: 5:30–6:30). Watching TV was the most common daily habit of the participants within one hour before bedtime, followed by using a smartphone or computer and engaging in relaxation activities.

Approximately half of the participants (51.2%) consumed coffee or tea after 4 p.m. Most of the participants (64.3%) reported no alcohol consumption in a typical week. Few participants (10.7%) reported smoking habits. Most of the participants (67.9%) were physically active for at least 40 min per day, which included doing housework or other work that involves physical activity, walking to work or school, cycling, doing fitness activities, or playing sports.

### 3.2. Bivariate Analysis of Mental Well-Being

The high-exposure community had the highest prevalence of poor mental well-being (38.2%, 95% confidence interval (CI) 29.8–46.6), followed by the low-exposure community (36.1%, 95% CI 27.1–45.1) and the no-exposure community (21.9%, 95% CI 15.4–28.4) (Table 2). There was a significant association between mental well-being and exposure level of the residential community (*p* = 0.005). There was also a significant association of mental well-being with perceived economic situation (*p* < 0.014), hearing ability (*p* < 0.001), chronic illness (*p* = 0.007), perceived level of noise (*p* < 0.001), physical activity (*p* = 0.001), and housing type (*p* = 0.019).

### 3.3. Bivariate Analysis of Sleep Disturbance

The high-exposure community had the highest prevalence of sleep disturbance (46.6%, 95% CI 38.0–55.2), followed by the low-exposure community (46.3%, 95% CI 36.9–55.7) and the no-exposure community (29.0%, 95% CI 22.0–36.0) (Table 3). There was a statistically significant association between sleep disturbance and residential community (*p* = 0.003). Additionally, there was a statistically significant association of sleep disturbance with perceived economic situation (*p* = 0.001), hearing ability (*p* = 0.011), chronic illness (*p* = 0.010), use of sleep medicines or supplements (*p* < 0.001), and perceived level of noise (*p* < 0.001).

### 3.4. Multivariate Analysis of Mental Well-Being

The bivariate logistic regression analysis with no adjustments for any covariates showed that residents in both the high- and low-exposure communities were significantly more likely to have poor mental well-being than the no-exposure community residents (odds ratio (OR): 2.01, 95% CI: 1.16–3.48; OR: 2.12, 95% CI: 1.31–3.70, respectively). In model 1, which adjusted for age and sex, the multivariate logistic regression analysis also showed that residents of both the high- and low-exposure communities were significantly more likely to have poor mental well-being than the residents of the no-exposure community (OR: 2.20, 95% CI: 1.31–3.70; OR: 2.04, 95% CI: 1.18–3.52, respectively; Table 4). In model 2, which adjusted for perceived economic situation, hearing ability, chronic illnesses, physical activities, sex, and age, the multivariate logistic regression analysis showed a similar significant association between residential community and mental well-being.

### 3.5. Multivariate Analysis of Sleep Disturbance

The bivariate logistic regression analysis with no adjustments showed that residents of both the high- and low-exposure communities were significantly more likely to have sleep disturbance than the residents of the no-exposure community (OR: 2.13, 95% CI: 1.31–3.47; OR: 2.11, 95% CI: 1.26–3.52, respectively). In model 1 (adjusted for age and sex), the multivariate logistic regression analysis showed that both the high- and low-exposure community were significantly more likely to have sleep disturbance than the no-exposure community (OR: 2.16, 95% CI: 1.32–3.52; OR: 2.18, 95% CI: 1.30–3.66, respectively; Table 5). In model 2, the multivariate logistic regression analysis showed a similar significant association between residential community and sleep disturbance.

## 4. Discussion

The main finding of this study was that residents living in communities exposed to noise from military aircraft were significantly more likely to report poor mental well-being than residents living in a non-exposed community. This finding suggests that the exposure to noise from the aircraft of Kadena Air Base led to poor mental well-being among people in the town of Kadena. A report on aircraft noise from Okinawa prefectural government in 1999 also showed that residents living in an area with high noise exposure reported lower life satisfaction than residents living in an area with low exposure or no exposure [12].

This finding is reasonable because it is biologically plausible. The most common response in areas exposed to environmental noise is annoyance [26], which indirectly affects mental health [27,28]. As mentioned in the introduction, almost all respondents to the community-based survey conducted in Kadena have reported feeling annoyance due to the loudness and various impacts of aircraft noise from Kadena Air Base [14].

This finding is in line with those of other studies that assessed the impact of military aircraft noise exposure on various health outcomes. A study conducted in the U.S.A used Geographic Information Systems (GIS)-based modeling of acoustic and flight data. The study predicted that within areas exposed to noise from military aircraft, tens of thousands of people are at risk of adverse health effects such as annoyance and sleep disturbance, and a significant number of people experience considerable annoyance [9]. A survey of residents living near military airfields in Japan also found that responses of annoyance to noise from military aircraft were substantially higher than the level predicted by exposure–response relationships for international civilian aviation [29]. The study highlighted that people living near military airfields in Japan experience disproportionately high annoyance from military aircraft noise compared to civilian aircraft and need context-specific health risk assessments. A study based on blood samples from 621 male air-force personnel exposed to intense noise comprehensively showed that chronic noise exposure significantly induces oxidative stress and inflammation, which contributes to physiological changes observed in hearing loss and other various biomarkers that impact overall health [30].

Our findings can be explained from the perspective of noise magnitude. Kadena Air Base covers 80% of the area of the town of Kadena and is adjacent to a residential community. The annual average noise levels in the noise-exposed communities in the present study have reached 53–64 dBA (*L_den_*) for many years [15,16,17] and significantly exceed the limit of 45 dBA (*L_den_*) recommended by the WHO’s noise guidelines for aircraft noise [2]. The WHO guidelines estimate that there is an absolute risk of 10% of the population becoming highly annoyed at a noise exposure level of 45.4 dBA (*L_den_*). Comparison of the exposure to aircraft noise at 50 dBA and 60 dBA has provided strong evidence of an association between aircraft noise and the percentage of the population highly annoyed for each 10-dBA increase (OR: 3.40, 95% CI: 2.42–4.80) [2]. However, these results do not include studies on noise from military aircraft.

A recent review paper on transportation noise, which does not consider military aircraft noise, and mental health on both humans and animals suggests that long-term exposure to decibel levels in the range of 50–70 dBA leads to activation of the sympathetic and endocrine systems, increases stress-hormone levels, and contributes to mental health conditions such as depression and anxiety [28]. A large case–control study with secondary data on health-insurance holders in Germany also reported a significant association between aircraft noise and depression, with an increased risk of depression being observed at aircraft noise levels of 50–55 dBA [31].

A study using census data in the United Kingdom showed that residents in high civil aircraft noise zones reported lower levels of well-being and relaxation, with consistent declines in subjective well-being occurring when noise exceeded 55 dBA [32,33]. Overall, the negative impact of aircraft noise on mental health has been consistently observed in areas where the levels are higher than the limit recommended by the WHO. Considering that these studies do not include the impact of noise from military aircraft, it is speculated that the effects in Kadena are even more severe.

Apart from the noise exposure, lack of physical activities was also associated with poor mental well-being in the study population. This finding is consistent with that of a study conducted in east Asian countries including Japan. The study reported that household physical activities were significantly associated with subjective well-being of older adult population [34]. Therefore, there is a possibility that promoting physical activities improves the well-being of people living near the bases in Okinawa.

Another second finding of this study was that residents living in communities exposed to noise from military aircraft were significantly more likely to report sleep disturbance than residents living in the non-exposed community. This finding suggests that exposure to continuous noise from military aircraft causes sleep disturbance among residents in communities with exposure to noise from Kadena Air Base. A report on the effects of aircraft noise in Okinawa indicated that residents in an area exposed to noise from Kadena Air Base reported sleep disturbance. Additionally, sleep disturbance was more frequent near Kadena Air Base than Futenma Air Station, suggesting that this difference may be related to the higher number of nighttime flights at Kadena Air Base than at Futenma Air Station [12].

This finding is biologically plausible in terms of the intensity of the noise. The WHO guidelines recommend limiting aircraft noise at night to below 40 dBA to prevent sleep disturbance [2]. In the noise-exposure communities investigated in the present study, the average nighttime noise levels were 40–49 dBA (*L_night_*). The frequency of nighttime flights increased from a monthly average of 166.2 in 2023 to 207.1 in 2024 and has continued to rise over the past five years (See Supplementary File S1) [15,16,17]. A study on 1005 adults living near a military airfield in South Korea found a clear exposure–response relationship between aircraft noise and poor sleep quality, with significantly higher prevalence of sleep disturbance in both low- and high-exposure groups than the control group [35].

A cross-sectional study on civilian aircraft in Poland using the same AIS as the present study also found that residents living in areas exposed to nighttime aircraft noise reported more sleep disturbance than residents living in non-exposed areas did (OR = 2.62; 95% CI: 1.01–6.89) [36]. A review of studies on environmental noise and sleep showed a negative correlation between exposure to aircraft noise and sleep disturbance [37,38]. Thus, there is a need for a more detailed study focusing on the sleep environment with the increased night flights.

The main strength of this study is the use of validated global scales. WHO-5 has been confirmed to be an easy and effective way to measure mental well-being for the general population [39] and is suitable for identifying depression in older adults [40]. The Japanese version has been validated and has demonstrated reliability [18,19,20]. Studies conducted in Japan using this scale found that the proportion of people aged 64 years and older with poor mental well-being ranged from 23.7% to 29.5%, regardless of noise exposure [20,41]. In the present study, 60% of the participants were over 60 years old, and the proportion of people with poor mental well-being in the community with no noise exposure was 21.9%. This is similar to the percentages obtained in the studies mentioned, indicating that our results are reliable.

AIS has also been validated for measuring sleep disturbance in several studies [23,24,25]. A large-scale Japanese community survey using AIS involved 7873 individuals under the National Health Insurance system comprising various workers, retired people, and unemployed people, and 23.4% of adults reported insomnia symptoms [42]. This value is similar to the prevalence of 29.0% for sleep disturbance in the no-exposure community in the present study, indicating that our results are reliable.

A major limitation of the present study was that the participation rate was lower than anticipated. As no information is available on the age, gender and other characteristics of non-participants, the impact of selection bias due to the non-response cannot be assessed.

Regarding the ORs of the associations between residential community and mental well-being and sleep disturbance, there are points to be discussed. First, the difference in the ORs between the high-exposure and low-exposure communities was small for both mental well-being and sleep disturbance. A possible reason for the small difference is the non-linear association between noise level and people’s responses [43]. Therefore, when noise level exceeds certain threshold, noise level could contribute less significantly to people’s well-being and sleep. Second, the ORs of the associations between residential community and mental well-being and sleep disturbance were around 2, suggesting that the strength of the associations was not so large. However, this does not mean that the effects of aircraft nose exposure in Okinawa are negligible. In 34 of the 36 noise-monitoring sites for the two U.S. air bases in Okinawa, the noise level exceeded 45 dBA (*L_den_*) in 2024 [15], which is the maximum noise level from aircraft recommended by the WHO to prevent negative health impacts. It was estimated in 1999 that approximately 480,000 people in Okinawa prefecture are exposed to aircraft noise exceeding environmental standards, which is equivalent to 38% of the prefecture’s population [12]. Considering the large number of people who are affected by long-term chronic exposure to military aircraft noise, this study confirms that there is an urgent need to address the noise from military aircraft in Okinawa.

## 5. Conclusions

This study showed that residents living in communities exposed to noise from military aircraft were significantly more likely to report poor mental well-being and sleep disturbance than residents living in no-exposed community. The results suggest that there is an urgent need to address the noise from military aircraft in Okinawa.

## Figures and Tables

**Table 1:** Characteristics of study participants (*n* = 394).

Characteristics			*n*	%
Residential community		No-exposure	155	39.3
		Low-exposure	108	27.4
		High-exposure	131	33.2
Sex		Male	174	44.2
		Female	220	55.8
Age range (years)		≤20 s	20	5.1
		30 s	23	5.8
		40 s	55	14.0
		50 s	64	16.2
		60 s	93	23.6
		70 s	100	25.4
		≥80 s	39	9.9
Employment type (*n* = 392)		Regular employee	88	22.4
		Housewife/husband	80	20.4
		Retiree	80	20.4
		Part-time job	65	16.6
		Self-employed	40	10.2
		Others	39	9.9
Number of household members (*n* = 390)		1	32	8.2
		2	140	35.9
		3	105	26.9
		≥4	113	29.0
Perceived economic situation		Very comfortable	6	1.5
		Comfortable	36	9.1
		So-so	245	62.2
		Difficult	78	19.8
		Very difficult	29	7.4
Hearing ability		No hearing difficulty	270	68.5
		Hearing difficulty	124	31.5
Use of hearing aids (*n* = 124)		No	110	88.7
		Yes	14	11.3
History of insomnia/other sleep problems (*n* = 388)		No	309	79.6
		Yes	79	20.4
Under treatment/observation (*n* = 74)		No	47	63.5
		Yes	27	36.5
Use of sleep medicines/supplements (*n* = 391)		No	338	86.4
		Yes	53	13.6
Perceived level of noise (*n* = 391)		Very noisy	105	26.9
		Noisy	79	20.2
		Slightly noisy	85	21.7
		Not bother	75	19.2
		Feel nothing/No aircraft noise	47	12.0
Hours of stay at/near home per day (*n* = 380)		<6 h	28	7.4
		≥6 and <12 h	69	18.2
		≥12 and <18 h	113	29.7
		≥18 and ≤24 h	170	44.7
Time of going to bed (*n* = 391); Median (interquartile range)			22:30 (21:30, 23:30)	
Time of waking up (*n* = 391); Median (interquartile range)			6:00 (5:30, 6:30)	
Napping (*n* = 390)		No	212	54.4
		Yes	178	45.6
Coffee/tea consumption after 4 p.m. (*n* = 389)		No	190	48.8
		Yes	199	51.2
Frequency of alcohol consumption per week (*n* = 392)		Do not drink	252	64.3
		1 to 3 times	72	18.4
		4 times or more	68	17.3
Smoking (*n* = 392)		No	350	89.3
		Yes	42	10.7
Physical activities (>40 min) per day (*n* = 390)		No	125	32.1
		Yes	265	67.9
Housing type (*n* = 393)		Single-family home	359	91.3
		Apartment	34	8.7
Years of residence; median (interquartile range)		22 (11, 37)		
Chronic illnesses (*n* = 388)		Absent	145	37.4
		Present	243	62.6
Chronic diseases/health condition *		Hypertension		150
		Cardiovascular diseases		74
		Chronic pain		65
		Endocrinology disease		41
		Hearing loss		23
		Respiratory disease		20
		Chronic itching		17
		Depression/mood disorders		18
		Others		41
Daily habits within one hour before going to bed *		Watching TV		266
		Using a smartphone/PC		151
		Relaxing		112
		Drinking alcohol		55
		Eating		35
		Smoking		17
		Exercising		10
		Bathing		7
		Others		18

* Multiple responses.

**Table 2:** Bivariate analysis of associations between participants’ characteristics and mental well-being (*n* = 394).

	Characteristics	Poor Well-Being		Moderate Well-Being		*p*-Value
		*n*	%	*n*	%	
Residential community						
	No-exposure	34	21.9	121	78.1	
	Low-exposure	39	36.1	69	63.9	
	High-exposure	50	38.2	81	61.8	0.005
Sex						
	Male	54	31.0	120	69.0	
	Female	69	31.4	151	68.6	1.000
Age						
	≤49 years	31	31.6	67	68.4	
	50–69 years	50	31.8	107	68.2	
	≥70 years	42	30.2	97	69.8	0.961
Employment type (*n* = 392)						
	Regular employee	28	31.8	60	68.2	
	Housewife/husband	34	42.5	46	57.5	
	Retirement	22	27.5	58	72.5	
	Part-time job	17	26.2	48	73.8	
	Self-employed	9	22.5	31	77.5	
	Others	11	28.2	28	71.8	0.199
Household members (*n* = 390)						
	1	7	21.9	25	78.1	
	2	44	31.4	96	68.6	
	≥3	69	31.7	149	68.3	0.569
Perceived economic situation						
	Comfortable/Very comfortable	9	21.4	33	78.6	
	So-so	69	28.2	176	71.8	
	Difficult/Very difficult	45	42.1	62	57.9	0.014
Hearing ability						
	No hearing difficulty	65	24.1	205	75.9	
	Hering difficulty	58	46.8	66	53.2	< 0.001
Chronic illnesses (*n* = 388)						
	Absent	34	23.4	111	76.6	
	Present	89	36.6	154	63.4	0.007
Sleep medicine/supplements (*n* = 391)						
	No	100	29.6	238	70.4	
	Yes	22	41.5	31	58.5	0.110
Perceived level of noise (*n* = 390)						
	Very noisy/Noisy/Slightly noisy	102	38.1	166	61.9	
	Not bothered/Feel nothing/No aircraft noise	21	17.2	101	82.8	<0.001
Daily hours at/near home (*n* = 380)						
	<18 h	66	31.4	144	68.6	
	≥18 h	56	32.9	114	67.1	0.825
Napping (*n* = 390)						
	No	60	28.3	152	71.7	
	Yes	63	35.4	115	64.6	0.155
Coffee/tea consumption (*n* = 389)						
	No	58	30.5	132	69.5	
	Yes	63	31.7	136	68.3	0.827
Alcohol consumption (*n* = 392)						
	Do not drink	85	33.7	167	66.3	
	Drink	38	27.1	102	72.9	0.211
Smoking (*n* = 392)						
	Yes	12	28.6	30	71.2	
	No	111	31.7	239	68.3	0.729
Physical activities per day (*n* = 390)						
	No	54	43.2	71	56.8	
	Yes	69	26.0	196	74.0	0.001
Housing type (*n* = 393)						
	Single-family home	105	29.2	254	70.8	
	Apartment	17	50.0	17	50.0	0.019

**Table 3:** Bivariate analysis of associations between participants’ characteristics and sleeping disturbance.

	Characteristics	Sleeping Disturbance		Non-Sleeping Disturbance		*p*-Value
		*n*	%	*n*	%	
Residential community						
	No-exposure	45	29.0	110	71.0	
	Low-exposure	50	46.3	58	53.7	
	High-exposure	61	46.6	70	53.4	0.003
Sex						
	Male	66	37.9	108	62.1	
	Female	90	40.9	130	59.1	0.604
Age						
	≤49 years	35	35.7	63	64.3	
	50–69 years	69	43.9	88	56.1	
	≥70 years	52	37.4	87	62.6	0.352
Employment type (*n* = 392)						
	Regular employee	31	35.2	57	64.8	
	Housewife/husband	39	48.8	41	51.3	
	Retirement	32	40.0	48	60.0	
	Part-time job	26	40.0	39	60.0	
	Self-employed	16	40.0	24	60.0	
	Others	10	25.6	29	74.4	0.246
Household members (*n* = 390)						
	1	11	34.4	21	65.6	
	2	58	41.4	82	58.6	
	≥3	85	39.0	133	61.0	0.748
Perceived economic situation						
	Comfortable/Very comfortable	15	35.7	27	64.3	
	So-so	82	33.5	163	66.5	
	Difficult/Very difficult	59	55.1	48	44.9	0.001
Hearing ability						
	No hearing difficult	95	35.2	175	64.8	
	Hearing difficulty	61	49.2	63	50.8	0.011
Chronic illnesses (*n* = 388)						
	Absent	46	31.7	99	68.3	
	Present	110	45.3	133	54.7	0.010
Sleep medicine/supplements (*n* = 391)						
	No	120	35.5	218	64.5	
	Yes	35	66.0	18	34.0	<0.001
Perceived level of noise (*n* = 390)						
	Very noisy/Noisy/Slightly noisy	134	50.0	134	50.0	
	Not bothered/Feel nothing/No aircraft noise	22	18.0	100	82.0	<0.001
Daily hours at/near home (*n* = 380)						
	<18 h	83	39.5	127	60.5	
	≥18 h	71	41.8	99	58.2	0.658
Napping (*n* = 390)						
	No	82	38.7	130	61.3	
	Yes	73	41.0	105	59.0	0.678
Coffee/tea consumption (*n* = 389)						
	No	76	40.0	114	60.0	
	Yes	78	39.2	121	60.8	0.918
Alcohol consumption (*n* = 392)						
	Do not drink	102	40.5	150	59.5	
	Drink	54	38.6	86	61.4	0.747
Smoking (*n* = 392)						
	No	140	40.0	210	60.0	
	Yes	16	38.1	26	61.9	0.869
Physical activities per day (*n* = 390)						
	No	47	37.6	78	62.4	
	Yes	108	40.8	157	59.2	0.581
Housing type (*n* = 393)						
	Single-family home	140	39.0	219	61.0	
	Apartment	15	44.1	19	55.9	0.585

**Table 4:** Multivariate analysis of association with mental well-being.

	Characteristics	Model 1		Model 2	
		AOR *	95% CI †	AOR *	95% CI †
Residential community					
	No-exposure	1.00	Reference	1.00	Reference
	Low-exposure	2.04	1.18–3.52	1.94	1.05–3.56
	High-exposure	2.20	1.31–3.70	1.84	1.05–3.23
Sex					
	Male	1.00	Reference	1.00	Reference
	Female	0.98	0.63–1.52	1.20	0.75–1.92
Age					
	≤49 years	1.00	Reference	1.00	Reference
	50–69 years	1.06	0.61–1.83	1.00	0.54–1.84
	≥70 years	0.94	0.54–1.67	0.67	0.33–1.33
Perceived economic situation					
	Comfortable/very comfortable			1.00	Reference
	So-so			1.30	0.56–3.02
	Difficult/very difficult			2.30	0.94–5.60
Hearing ability					
	No hearing difficulty			1.00	Reference
	Hearing difficulty			2.62	1.56–4.42
Chronic illnesses					
	Absent			1.00	Reference
	Present			1.96	1.14–3.36
Physical activities					
	No			1.00	Reference
	Yes			0.39	0.24–0.64

*: adjusted odds ratio; †: confidence interval.

**Table 5:** Multivariate analysis of association with sleep disturbance.

	Characteristics	Model 1		Model 2	
		AOR *	95% CI †	AOR *	95% CI †
Residential community					
	No-exposure	1.00	Reference	1.00	Reference
	Low-exposure	2.18	1.30–3.66	2.04	1.16–3.59
	High-exposure	2.16	1.32–3.52	1.98	1.17–3.35
Sex					
	Male	1.00	Reference	1.00	Reference
	Female	1.10	0.73–1.67	1.12	0.72–1.76
Age					
	≤49 years	1.00	Reference	1.00	Reference
	50–69 years	1.50	0.89–2.55	1.44	0.81–2.56
	≥70 years	1.09	0.63–1.88	0.82	0.43–1.57
Perceived economic situation					
	Comfortable/very comfortable			1.00	Reference
	So-so			0.73	0.36–1.51
	Difficult/very difficult			1.87	0.86–4.09
Hearing ability					
	No hearing difficulty			1.00	Reference
	Hearing difficulty			1.34	0.81–2.22
Chronic illnesses					
	Absent			1.00	Reference
	Present			1.64	0.99–2.71
Sleep medicines/supplements					
	Used			1.00	Reference
	Not used			2.95	1.54–5.64

*: adjusted odds ratio; †: confidence interval.

## Data Availability

Data are available from the corresponding author upon reasonable request.

## Supplementary Materials

The following supporting information can be downloaded at: https://www.mdpi.com/article/10.3390/ijerph23010054/s1, File S1: Summary of annual average noise data (dBA *) at each measurement point in Kadena town. This table was extracted from “Summary of Aircraft Noise Measurement Results, Kadena Air Base and Futenma Air Station” reported by the Okinawa prefectural government over the past five years (2020–2024) and translated into Japanese by the authors.
