# Supplementary material for: Association of Military Aircraft Noise Exposure with Mental Well-Being and Sleep Disturbance near a Military Air Base in Okinawa, Japan: An Ecological Study
Source: Int J Environ Res Public Health. 2025 Dec 31;23(1):54. doi: 10.3390/ijerph23010054 (PMC12841551; doi:10.3390/ijerph23010054)
Supplement: Supplementary file 1 [file ijerph-23-00054-s001.zip › ijerph-3990006-supplementary.pdf]

Supplementary file 1: Summary of annual average noise data (dBA*) at each measurement point in Kadena town. This table was extracted from "Summary of Aircraft Noise Measurement Results, Kadena Air Base and Futenma Air Station" reported by the Okinawa prefectural government over the past five years (2020-2024) and translated into Japanese by the authors.

Measurement point		2020	2021	2022	2023	2024
Yara A (a point within the high-exposure community)	Average *Lden*	60	60	61	64	63
	Average number of noise occurrence/day	60.2	58.7	66.4	65.4	70.6
	Average *Lnight*	47	48	45	45	47
	Average number of noise occurrence in night†/month	89.0	136.1	133.9	166.2	207.1
Yara B	Average *Lden*	60	60	61	63	57
	Average number of noise occurrence/day	35.2	32.9	37.8	34.1	21.2
	Average *Lnight*	48	49	46	46	40
	Average number of noise occurrence in night/month	45.0	62.7	43.3	37.1	15.8
Kadena A	Average *Lden*	62	63	60	63	61
	Average number of noise occurrence/day	39.6	41.0	38.5	38.1	32.5
	Average *Lnight*	53	54	48	48	49
	Average number of noise occurrence in night/month	96.1	129.7	63.3	74.2	61.2
Kadena B	Average *Lden*	67	66	63	64	63
	Average number of noise occurrence/day	57.9	47.1	45.2	44.7	38.5
	Average *Lnight*	57	56	51	50	50
	Average number of noise occurrence in night/month	136.0	156.4	105.3	108.2	94.5
Kaneku (a point within the low-exposure community)	Average *Lden*	57	57	57	57	61
	Average number of noise occurrence/day	20.6	21.0	24.7	23.4	27.5
	Average *Lnight*	46	46	42	41	48
	Average number of noise occurrence in night/month	22.6	25.5	17.4	13.8	28.2

*: Noise level (dBA) was automatically recorded throughout the year using noise measurement equipment installed outdoors and averaged by being divided by the number of measurement days.

†: From 10 PM to 7 AM the following morning.
